# An inexpensive device to treat postpartum hemorrhage: a preliminary proof of concept study of health provider opinion and training in Nepal

**DOI:** 10.1186/1471-2393-14-81

**Published:** 2014-02-24

**Authors:** Nancy L Kerr, Mark Hauswald, Suman Raj Tamrakar, David A Wachter, Gillian M Baty

**Affiliations:** 1 Department of Obstetrics and Gynecology, University of New Mexico School of Medicine, 1 University of New Mexico, Albuquerque, NM 87131, USA; 2 Department of Emergency Medicine, University of New Mexico, MSC 11 6025, 1 University of New Mexico, Mexico 87131, USA; 3 Department of Gynecology and Obstetrics, Kathmandu University School of Medical Sciences, Dhulikhel, Kavre, Nepal

**Keywords:** Aortic compression, Anti-shock garment, Maternal mortality, Military anti-shock trousers, Nepal, Pelvis/blood supply, Postpartum hemorrhage/therapy

## Abstract

**Background:**

Obstetric hemorrhage remains the leading cause of maternal mortality in resource limited areas. An inexpensive pneumatic anti-shock garment was devised of bicycle tubes and tailored cloth which can be prepared from local materials in resource-limited settings. The main purposes of this study were: 1) to determine acceptability of the device by nurses and midwives and obtain suggestions for making the device more suitable for use in their particular work environments, 2) to determine whether a three hour training course provided adequate instruction in the use of this device for the application of circumferential abdominal pelvic pressure, and 3) determine production capability and cost in a resource-limited country.

**Methods:**

Fifty-eight nurse and midwife participants took part in three sessions over eight months in Nepal. Correct device placement was assessed on non-pregnant participants using ultrasound measurement of distal aortic flow before and after device inflation, and analyzed using confidence intervals. Participants were surveyed to determine acceptability of the device, obtain suggestions for improvement, and to collect data on clinical use.

**Results:**

Device placement achieved flow decreases with a mean of 39% (95% CI 25%-53%, p < 0.001) in the first session, 28% (95% CI 21%-33%, P < 0.001) after four months and 29% (95% CI 24%-34%, p < 0.001) at 8 months. All nurses and midwives thought the device would be acceptable for use in obstetric hemorrhage and that they could make, clean, and apply it. They quickly learned to apply the device, remembered how to apply it, and were willing and able to use the device clinically. Ten providers used the device, each on one patient, to treat obstetric hemorrhage after routine measures had failed; bleeding stopped promptly in all ten, two of whom were transported to the hospital. Production of devices in Kathmandu using local tailors and supplies cost approximately $40 per device, in a limited production setting.

**Conclusions:**

Preliminary data suggest that an inexpensive, easily-made device is potentially an appropriate addition to current obstetric hemorrhage treatment in resource-limited areas and that further study is warranted.

## Background

Obstetric hemorrhage is the leading cause of maternal death worldwide, including 33.9% of cases in Africa and 30.8% in Asia [1]. Most cases are postpartum and initial treatment includes uterotonic medications such as oxytocin and misoprostol plus bimanual massage. However, in rural and remote areas, these drugs are often not available. When bleeding is not due to atony or is not responsive to these interventions, other approaches are needed. Treatment is most effective when initiated promptly, so it needs to be readily available at the birth site -- which in many resource-limited areas is the home or a small clinic. Aortic compression is an age-old method for treatment of obstetric hemorrhage (or pelvic trauma) using an open hand, fist, or knee to compress the aorta against the underlying lumbar vertebrae [2,3]. Prolonged compression is difficult, however, when personnel are limited or other interventions or transport are needed. Anti-shock garments provide circumferential abdominal-pelvic pressure (CAPP), which incorporates the essence of aortic compression. They can be pneumatic (inflatable) anti-shock garments (PASG) or non-inflatable anti-shock garments (NASG) in design. Their use for postpartum hemorrhage is not new and there are numerous case reports of successful use of PASG and NASG for obstetric and post-operative hemorrhage [4]. Two pre-intervention/intervention trials with NASG showed improved maternal outcomes [5]. A cluster randomized trial of NASG for obstetrical hemorrhage was promising although it did not reach statistical significance [6].

Pneumatic anti-shock garments (also called Military or Medical Anti-Shock Trousers) were developed in the early 1900’s to treat shock in surgical and battlefield patients. They were used extensively to treat trauma patients in the United States until the mid-1980’s, when studies revealed slightly decreased survival rates in a population of urban pre-hospital trauma patients with quick transport to a trauma center and suffering primarily from injuries above the pelvis [7]. To some degree the term “anti-shock” may be a misnomer because the devices only squeeze about 250 ml of blood from the pelvis and legs to the upper body [8]. However, they do greatly decrease pelvic perfusion by delivering direct pressure to pelvic compartment organs and to smaller pelvic blood vessels. Commercial pneumatic anti-shock garments have been shown to decrease distal aortic blood flow by up to 90% [9] at the level of the superior mesenteric artery. The NASG was shown to decrease distal aortic blood flow by about one-third (34%) [10]. PASG and NASG can be used while additional treatment is being performed or prepared for and can provide definitive treatment in some cases if they provide complete hemostasis. PASG are extremely safe, with an estimated complication rate of less than one per 100,000 cases [11]. Unfortunately, commercial PASG are relatively expensive and both devices are bulky and relatively difficult to clean. We believe that there is a need for an inexpensive aortic compression/anti-shock device that can be made almost anywhere in the world and be easily available at all births, at home or in a health care facility. We therefore designed a new PASG which can be improvised on the spot from bicycle tubes and cloth sheets, or prepared in advance by a local tailor or seamstress. We elected to call this a CAPP (circumferential abdominal-pelvic pressure) device to clarify its mechanism of action and differentiate it from commercial PASGs. Our prototype CAPP device utilized three bicycle tubes, one placed over each leg and one coiled on the lower abdomen; each was then tightly over-wrapped with bed sheets and secured with adhesive tape. We found that, when applied by physicians on healthy non-pregnant volunteers in the United States, this device decreased distal aortic blood flow by over half (56%) [10].

Nepal was selected for several reasons. The overall maternal mortality rate is high, at 240 per 100,000 live births (uncertainty interval 149–370) in 2008 [12]. The percentage of births attended by a skilled provider is low (36%), and the percentage of deliveries occurring in a facility (hospital, primary health center, health post or sub-health post) was only 28% during the five years prior to the 2011 Demographic Health Survey [13]. At health and sub-health posts births are attended by nurses or midwives (auxiliary nurse midwives) and surgery and blood transfusion are not available. Midwives are also called upon to attend home deliveries, particularly when problems occur. Transportation of patients is difficult in Nepal due to topography and lack of infrastructure. Nurses perform the majority of in-hospital uncomplicated vaginal deliveries in Nepal. Nurses receive 36 months of nursing education after 12 years of basic education. Auxiliary nurse midwives receive 18 or 24 months of training after at least 8 years of basic education.

**Figure 1:**
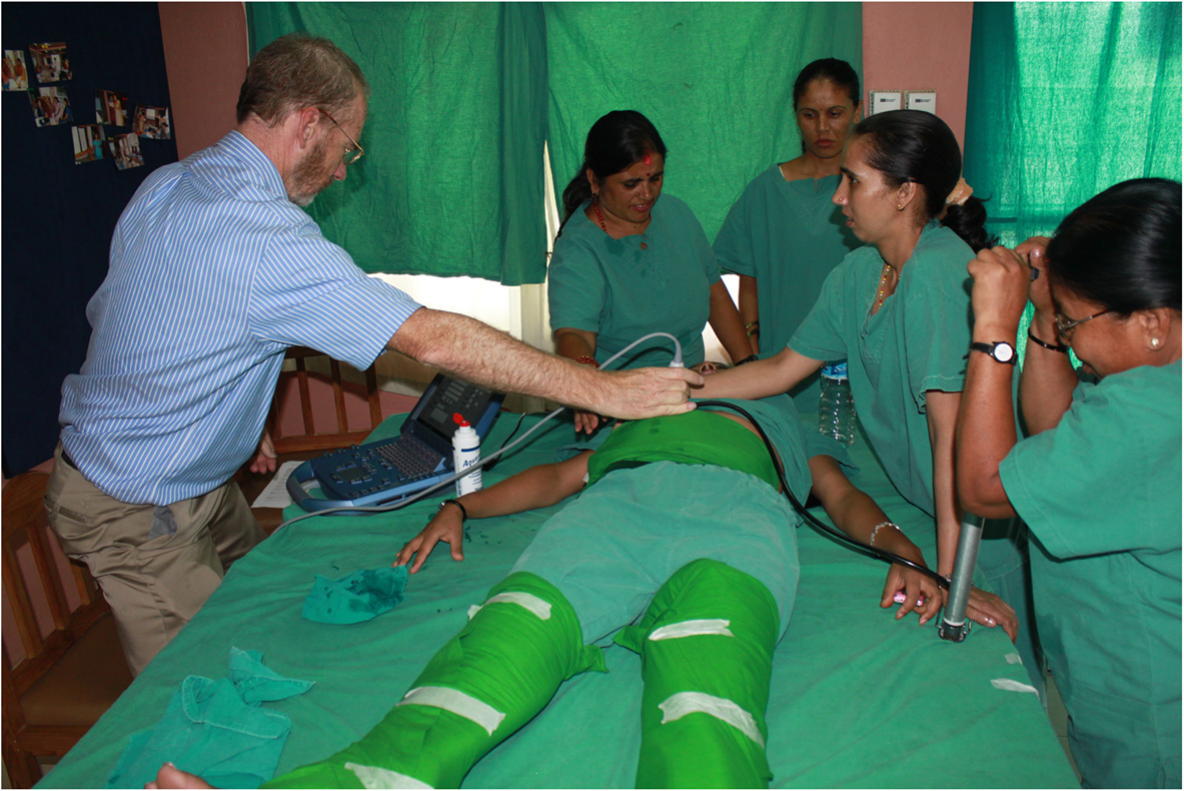
CAPP device tailored from sheet material; pockets contain each bicycle tube, inflated.

**Figure 2:**
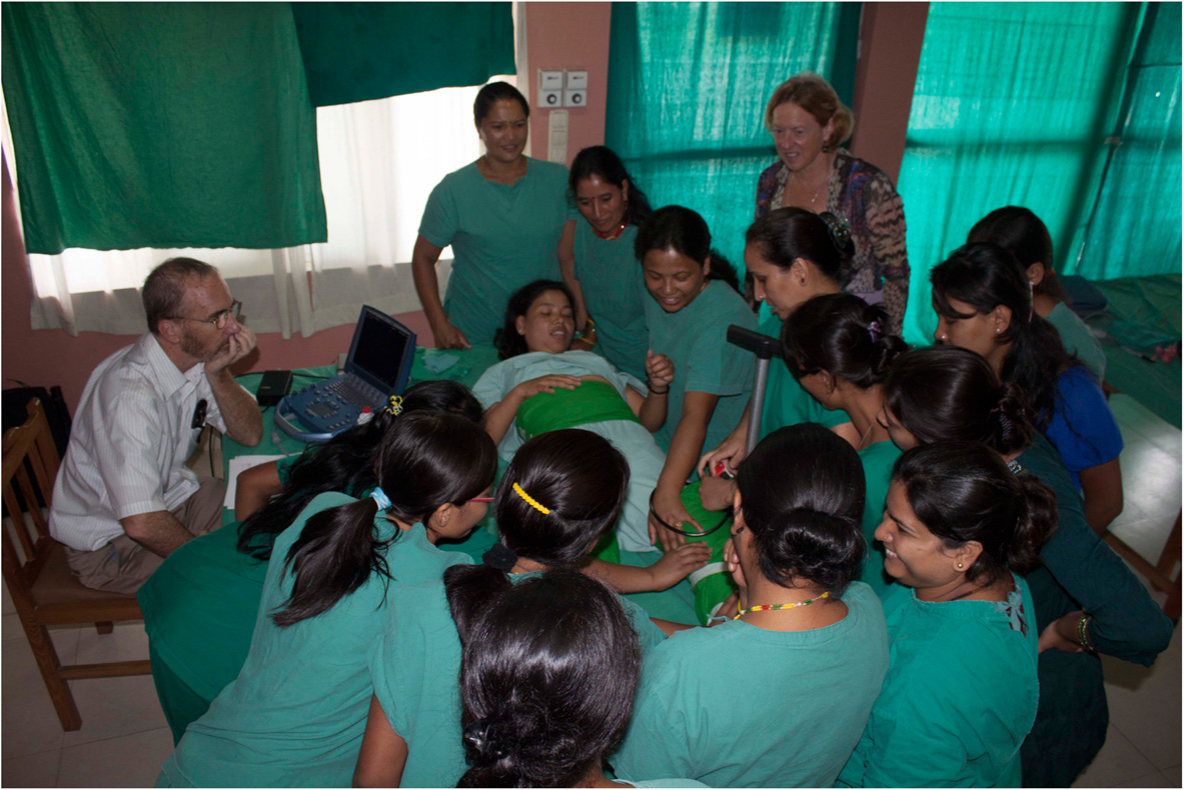
Class demonstration of device application with nurses and midwives.

**Figure 3:**
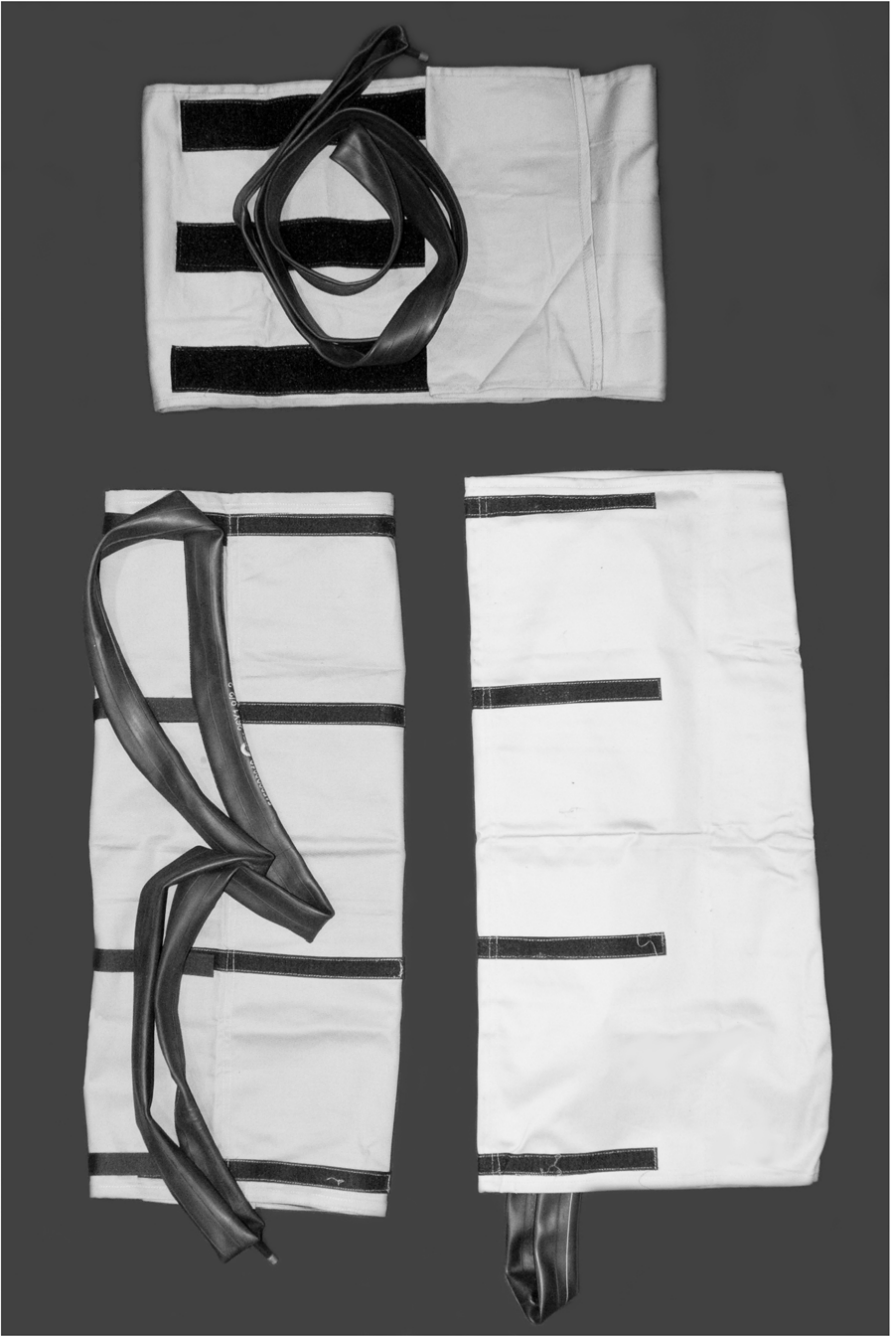
**CAPP device with hook-and-loop (Velcro™) on abdominal and leg pieces.**

During preliminary discussions at Dhulikhel Hospital, Kathmandu University School of Medical Sciences in 2010, staff nurses suggested that modification was needed to expedite application by practitioners working “solo” in small facilities and homes. Accordingly, the current study used a modified device with tailored abdominal and leg compartments with pockets to contain the bicycle tubes, which are inflated with a bicycle pump after the compartments are secured in place (Figures 1 and 2). The final version was further modified to include utilized hook-and-loop strips (Velcro™) for closure instead of tape (Figure 3). All components, including fabric, bicycle tubes and pumps, were purchased in Kathmandu and tailored locally. Total cost per device was approximately US $40, of which about half was for the pump and bicycle tubes. The device’s cloth elements can be laundered like hospital sheets, and the inflatable parts washed or cleaned by soaking in mild bleach solution.

Diffusion of innovative devices and treatments can be difficult and slow unless they meet a clear need for the intended user, are relatively easy to obtain and use, and are culturally acceptable [14]. The objectives of the current study were to determine whether obstetrics providers in a resource-limited setting believe this inexpensive tailored CAPP device would be clinically useful and acceptable, and whether they would be willing and able to use it.

## Methods

The study was done over three sessions at Kathmandu University during August 2011, November 2011 and April 2012. The first was primarily a training session in use of the CAPP device. The second and third were primarily evaluative and intended to elicit attitudes and measure skill maintenance over time.

The study enrolled 58 participants including nurses and midwives in Kavre and neighboring districts, who represented a cross-section of non-physician skilled birth attendants. Midwives were recruited from a variety of small health facilities including sub-health posts. Many came from rural areas that were up to a two days’ walk from the nearest road. We attempted to include representatives from the various kinds of facilities available to pregnant women in Nepal. Inclusion criteria were all active midwives and all nurses who had completed Skilled Birth Attendant (SBA) training and were hence eligible to provide midwifery services. Participants were randomly selected by name from all the 344 primary health posts, health posts and subhealth posts that are in the catchment area for Kathmandu University Dhulikhel Hospital. This hospital, which is the *de facto* district hospital for Kavre District also serves as the obstetric tertiary care center for four neighboring rural districts: Sindhupoalchowk, Dolakha, Ramechhap and Sindhuli. Midwives and nurses were contacted through the appropriate District Health Office which is the governing body for health for each district. Ramechhap District Hospital refers tertiary care obstetric patients to Dhulikhel Hospital and there is one small private hospital slightly closer to Kathmandu, Scheer Memorial Hospital, which also serves as a referral facility for a limited number of obstetric patients. Both hospitals were included. The number of providers from each facility was limited to that which could be away from the facility without impacting patient care, with a goal of recruiting approximately sixty participants from a representative variety of facilities. Sixty was the estimated number needed by the research protocol. Three hospitals, eight primary health centers, thirteen health posts, nine sub health posts and two Dhulikhel Hospital outreach centers were eventually included.

Participants received reimbursement for travel, food and lodging, but were not compensated for their time. Initial training involved bringing all participants, in five groups of 10 to 15, to Dhulikhel Hospital. A one hour review of postpartum hemorrhage management was given in English and Nepali. The device was demonstrated and each participant learned to place it on a partner, with a third participant assisting. It was stressed that the device was to be used in conjunction with standard methods of treatment, which generally included oxytocin and/or misoprostol, uterine massage, and manual removal of the placenta or placental fragments if needed. Detailed information about study format and potential risks and benefits of the study were presented. Participants who were pregnant, had abdominal complaints or declined having it placed were excluded from having the device used on upon them, but could place it on others.

Quantitative information was obtained using a written survey instrument specifically designed to elicit feedback covering the listed objectives from this study population. Participants were asked six questions which had yes/maybe/no response options (reproduced verbatim in Table 1). One additional, open-ended question: “Do you have specific concerns or suggestions about the CAPP device?” elicited qualitative data. This question was first asked individually using the written form and later discussed in a small group session to allow “brainstorming” among participants. The surveys were in English, the standard language for medical instruction in Nepal, and each participant also reviewed her responses with a bilingual Nepali obstetrician (SRT) to allow her to clarify any potentially ambiguous or incomplete responses.

**Table 1:** Participant attitudes toward the CAPP device across three training sessions

	**Session 1**			**Session 2 (4 mo.)**			**Session 3 (8 mo.)**		
	**n = 58**			**n = 51**			**n = 50**		
	**Yes**	**Maybe**	**No**	**Yes**	**Maybe**	**No**	**Yes**	**Maybe**	**No**
Do you feel comfortable that you could successfully apply the CAPP device?	58	0	0	50	1^a^	0	50	0	0
Do you think that the CAPP device would help treat postpartum hemorrhage?	58	0	0	51	0	0	**	**	**
If you had the CAPP device would you use it to help treat postpartum hemorrhage?	58	0	0	51	0	0	50	0	0
Do you think that you could successfully clean a CAPP device?	58	0	0	51	0	0	**	**	**
Would you make a CAPP device if plans were available?	58	0	0	51	0	0	**	**	**
Have you used a CAPP device clinically on a patient?	*	*	*	9	*	42	10^b^	*	40

*Not applicable.

**Not asked.

^a^One provider thought she would need help from “the patient’s sister”.

^b^Includes the 9 patients at 4 months.

To assess their skill in device placement, the partner’s distal aortic blood flow below the takeoff of the superior mesenteric artery was measured using ultrasound, first at baseline and then with the device inflated. This quantitative data was used to confirm that participants had learned the psychomotor skills needed to use the device competently.

Participants were given one device “kit”, including tailored cloth pieces, bicycle tubes, and pump. They were told that they could use the device if they believed it was needed, but only after all standard treatments had been initiated.

The assessments were repeated during the second session at four months post training and during the third session at eight months. Each participant again used the device on a partner and placement was assessed by ultrasound measurement of distal aortic flow. The same basic survey was administered (Table 1), and those who had used the device clinically were individually given an additional “clinical use” survey that asked about the circumstances and outcome and was followed by a personal interview with a Nepali-speaking physician-researcher (SRT).

Fifty-eight participants were enrolled and completed the first session. At the four month session, fifty one participants returned. Of the seven participants who did not return, one who had been pregnant had delivered, one left the district for further education, two needed to attend to ill family members, and three were from a distant health-post that could not be contacted. Fifty participants returned at eight months; the one who did not return had delivered in the interim.

The University of New Mexico’s Institutional Review Board (IRB) ruled this study exempt under Federal Regulation 45CFR46.101(b) on 6/30/2010 (HRC #10-229) and approval for this study was obtained from the IRB of Kathmandu University School of Medical Sciences in Dhulikhel, Nepal on 6/24/2011 (#11-11). Studies of educational methodology do not require written informed consent and it was not required by either IRB. Participants were free to opt out of the educational program or to quit at any time. There was no direct benefit to them for participating and there was no disadvantage to withdrawal. All participants did provide written consent to be photographed for the figures.

Although this study was not a clinical trial it was registered at clinicaltrials.gov (identifier: NCT01497756) to track any complications or adverse events occurring with clinical use of the CAPP device. PASG are an approved treatment modality classified as pre-amendment devices under FDA regulations and have been extensively marketed and used for pelvic bleeding, so it was deemed to be impossible and unethical to prohibit use as a last resort in the clinical setting. However, the researchers and IRBs felt that failure to track clinical use would potentially put future patients at risk if complications were not identified and reported.

Measurements of aortic flow were recorded on paper and electronic memory cards from the ultrasound machines (SonoSite MicroMaxx™). Data were transferred using a double data entry format to an Excel™ (Microsoft Corporation, Redmond WA) spreadsheet. The decrease in distal aortic flow was calculated using Excel™. Stata 11® (StataCorp LP, College Station, Texas) was used for statistical analysis. Survey data were recorded numerically when possible and descriptively when appropriate.

## Results

At the initial training the mean reduction in distal aortic flow with application of the device was 39% (95% CI 25% - 53%, P < 0.001). At the four month session mean blood flow reduction was 28% (95 CI 21% - 33%, P < 0.001). At the eight month session mean distal aortic flow reduction was 29% (95% CI 24% - 34%, P < 0.001).

Upon being surveyed, all participants believed they were capable of tailoring, assembling, and cleaning the device and that it would be helpful for treatment of postpartum hemorrhage patients. All participants believed they could successfully apply it alone, but one thought she would need help from “the patient’s sister”.

Ten participants, all from health or sub-health posts, used the device clinically, each on a single patient. Bleeding promptly stopped and all ten patients had good outcomes. There were no adverse effects except that one patient requested prompt removal of the device after her bleeding stopped because of leg pain. Two patients were transported with the device in place to a hospital for further care, one for a massive vulvar-vaginal hematoma and another for a delayed postpartum hemorrhage ten days post-delivery.

Participants made several suggestions for modification of the device. In response, an additional small group compared a 20 cm diameter inflatable ball (standard soccer ball size) to the bicycle tube as the inflatable portion of the abdominal piece. They noted the ball to be quicker and easier to place and inflate than the bicycle tube, and more comfortable for the wearer.

## Discussion

The three devices that apply circumferential abdominal-pelvic pressure have different potential advantages and disadvantages. Commercial PASG have a long track record of successful use by minimally trained personnel as well as physicians [11]. The device is considerably better at decreasing pelvic blood flow than the alternatives [9]. It seems logical that commercial PASG be used in hospital facilities for management of obstetric hemorrhage to acutely decrease blood loss while resuscitating the patient. This would allow more aggressive options such as uterine artery embolization, laparotomy with hemostatic sutures or hysterectomy, if still needed, to be done on a more stable patient. However, commercial PASG are relatively expensive, complex in design and manufacture, difficult to repair and awkward to clean. This makes them less suitable for smaller facilities in low resource settings.

NASG are much less effective at decreasing pelvic perfusion [10] but are less expensive. They are the least complex of the three devices. Manufacture and repair are considerably easier than for commercial PASG although they still require specialized supplies and equipment. They are also easier to clean than commercial PASG. They are made of neoprene which degrades very slowly when stored but much more rapidly in the presence of petroleum products or bleach. NASG have been successfully used to treat obstetric hemorrhage in low resource environments [5].

Our CAPP/non-commercial PASG device is more effective at decreasing pelvic perfusion than NASG but less effective than commercial PASG [10]. It is easy to clean. It is as complex in design as the commercial PASG but can be made and repaired by anyone with a sewing machine and access to suitable cloth, tape or hook and loop closures, bicycle tubes and a pump. Local production can contribute to local economies and open opportunities for locally organized trainings and social marketing. Birth attendants can potentially make their own. It is the least expensive of the three devices and its modular design allows for replacement of individual parts if necessary. These characteristics mean that this CAPP device potentially has a much shorter supply chain than the other devices and that it can be easily replaced if, for example, it is used on a patient who is transported to distant facility. The pressure in both our device and in commercial PASG can be varied while they are being used, making it possible for the provider to titrate pressure as needed to control hemorrhage while minimizing discomfort.

All three devices are easy to use and require minimal training. They all work – i.e. they decrease pelvic blood flow. They are all safe – even the 90% decrease in pelvic blood flow from commercial PASG is tolerated well and is associated with an extraordinarily low complication rate. Given that obstetric hemorrhage is to a large degree unpredictable, treatment is most effective before complications occur, and the devices are very safe, it seems best to have them available at every birth, including home births, so that early application is possible. In facilities with a high volume of deliveries, an adequate supply of devices would allow them to be used more frequently and earlier before hemorrhage becomes severe and shock ensues.

There are limitations to this study. The CAPP device was applied to healthy, non-pregnant women who were not immediately postpartum, volume depleted, or in hemorrhagic shock, and therefore the decreases in flow may not be predictive of absolute flow change in patients with obstetric hemorrhage. Blood flow in the distal aorta is not identical to uterine artery flow. Additionally, the optimal amount of blood flow reduction is not known and is unlikely to be the same for all patients. The study was not designed to directly evaluate patient acceptability or clinical effectiveness.

## Conclusions

In summary, nurses and midwives in Nepal were quickly able to learn to use an inexpensive CAPP/non-commercial PASG device with the educational methodology presented and were enthusiastic about its use. They believed that they could make and maintain the device themselves. Participants were able to decrease distal aortic flow in healthy non-pregnant volunteers by about one third, with significant changes persisting for at least eight months after training. The limited clinical experience was uniformly positive. Our preliminary data suggest that this device is likely to be at least as effective as NASG and can be easily made inexpensively in a resource-limited country. Devices which decrease pelvic perfusion complement other methods for treatment of obstetric hemorrhage and may facilitate transport of the patient to a higher level of care in more stable condition.

We believe that this inexpensive CAPP device is a potentially valuable addition to management options for obstetric hemorrhage and propose that further study be done to assess its clinical efficacy as an addition to integrated strategies for decreasing maternal mortality [15].

## Abbreviations

CAPP: Circumferential abdominal-pelvic pressure; IRB: Institutional Review Board; MAST: Military or medical anti-shock trousers; NASG: Non-inflatable anti-shock garment; PASG: Pneumatic anti-shock garment.

## Competing interests

Four of the authors are employed by the University of New Mexico. STC. UNM, which is an affiliate of the University of New Mexico, has a patent pending for the CAPP device, but has agreed not to charge patent fees for its non-profit production.

## Authors’ contributions

NLK and MH developed the CAPP device and the initial concept for the study. NLK, MH, SRJ, DAW and GMB refined the study design and carried out the study including teaching and data collection. NLK and MH co-wrote the manuscript drafts and all authors read, provided substantial input to, and approved the final manuscript.

## Pre-publication history

The pre-publication history for this paper can be accessed here:

http://www.biomedcentral.com/1471-2393/14/81/prepub
